# The illusion of universality: The use of Nordic population registers in studies of migration, employment and health

**DOI:** 10.1177/1403494820945919

**Published:** 2020-08-10

**Authors:** Karl Gauffin

**Affiliations:** Department of Public Health Sciences, Stockholm University, Sweden

**Keywords:** Nordic registers, precarious employment, migration, Nordic welfare state, employment and health

## Abstract

*Aims:* Nordic register material is often considered to be a gold standard for studies of social epidemiology and population health, but it comes with certain limitations. This short communication aims to draw attention to lacking coverage as a potentially growing problem of Nordic register material. *Methods:* The article is based on a short review of previous studies and commentaries on the strengths and limitations of Nordic register data with a particular focus on studies of employment and migration. *Results:* In times of institutional and demographic change in the Nordic countries, the assumption of universal register coverage can be challenged. Precarious and informal employment arrangements, important social determinants of health, provide a good illustration of the problem. Work that is carried out in the semi-legal margins of the labour market, sometimes by a ‘hidden population’ of non-resident, short-term labour immigrants, will not be covered by the registers. Researchers may therefore run the risk of misrepresenting reality if they maintain the belief that population registers cover the entire population. ***Conclusions:* The Nordic registers are an extraordinary resource for public health researchers, but continuous quality control and assessment of validity and completeness will be crucial to maintain relevance in a transitioning society.**

## Strengths and limitations of a unique population material

For good reasons, the *Scandinavian Journal of Public Health* has dedicated three issues to the use of Nordic register data [1]. The population registers offer researchers in the Nordic countries a number of extraordinary advantages and are often a cause of both astonishment and envy in the international public health research community. There are at least three features of Nordic registers that make them stand out in comparison with other large population data materials. First, the possibility of record linkage allows for rich datasets combining individual information on employment, education, social insurances, criminality and health. Second, there is unmatched potential for life-course and intergenerational studies provided by lifelong follow-up time and data linkages between parents and offspring. Finally, because a personal identification number is the sole basis for register inclusion, the registers have long been assumed to offer something that survey-based material could never achieve: close to universal coverage of the entire national population [2].

These tremendous benefits aside, register research also comes with some challenges. A main point of concern relates to the heavy and highly complex nature of data collection and administration, which underlines the importance of data quality control. Validity and completeness were appropriately highlighted as a main concern in this article series, with both the editors and a number of study authors drawing attention to these problems and making some helpful distinctions. Validity can be seen as a way to express that what is stored in the database is also true in the world (true cases in the register divided by the total number of cases in the register). Completeness, on the other hand, means that what is true in the world is also stored in the database (true cases in the register divided by the actual number of true cases) [3,4].

## Problems of measurement

Register shortfalls in terms of validity and completeness are traditionally attributed to inaccuracies in measurement. For example, in a study published in the first issue of this article series, the authors relate their eye-opening finding that one third of drug-related deaths were not captured by the Swedish death certificates due to the complexity of drug-related mortality as well as the lacking precision of the death certificates and the ICD-codes used [5]. Another obvious limitation refers to the inability of registers to make detailed measures of individual perceptions of reality. Even though registers deliver excellent data on health and employment, they fail to capture individuals’ subjective evaluation of personal well-being or working environment, with researchers instead using proxy measures of such phenomena. For example, register data on medical care is always going to be a proxy measure of ill health, given the fact that many cases of sickness will never be subject to treatment. Records of unemployment, social insurances, income development and occupational classification can be used to give an estimation of employment quality, but given the missing data on other central aspects, such as work environment or type of employment contract, the measure will always be imperfect in the sense that it does not give information on the lived experience of such a condition. These examples illustrate problems of measurement but not of coverage. In other words, the missing completeness of registers is usually attributed to flawed measures that fail to signify a true case as such. Sampling bias, on the other hand, is usually not considered to be a problem for the Nordic registers, given the assumption of universal coverage based on personal identification numbers. However, recent developments in the Nordic countries might give reason to call this assumption into question.

## The illusion of universality

The limitations of register coverage may be illustrated by the topic of precarious employment in the Nordic countries. Sometimes described as ‘atypical’ or ‘nonstandard’ working arrangements, precarious employment commonly refers to low wage labour with high employment insecurity and lack of rights and protections [6]. The present-day form of precarious employment is often studied in relation to institutional and demographic developments and has also been presented as a central social determinant of health [7,8]. This combination of factors invites researchers in the Nordic countries to make use of population registers to explore the connections between precarious employment, migration and health [9]. Such attempts will quickly discover the limitations regarding measurement, as central dimensions of precarious employment cannot be captured by administrative data. Here, the assumption of universal coverage may be regarded as a strength compensating for these limitations in measurement [10].

However, when labour is carried out beyond the scope of registers, this assumption of complete coverage can, and should be, questioned. Precarious work may take place in the legal grey zones of the labour market, partly because individual workers in the informal work economy are easier to control and exploit compared to organised worker collectives, in particular if they are immigrants and depend on their employers to retain residency and other rights. This makes immigrants a particularly important group to consider when studying the development of a ‘hidden population’ performing precarious work in the Nordic countries.

Furthermore, administrative regulations on employment registration may create additional gaps in the Nordic employment statistics. In Sweden, short-term labour immigrants from European countries are not required to register as residents. In fact, the condition for a formal registration, which is the basis for inclusion in most national registers, is an estimated residence of 12 months or more. Because of this, information on labour immigrants’ individual employment conditions are not available, but aggregate information can be deduced from employer income statements. Statistics Sweden estimates that employment among non-resident workers increased 131% from 1997 to 2016, compared with a 16% increase among resident workers [11]. The strongest relative increase was found in low-wage occupational sectors, including catering, construction work and cleaning services. This suggests that Swedish register-based research may miss an increasing proportion of precarious work carried out by non-registered labour immigrants. In addition, the high number of denied asylum applications in recent years will likely lead to an increasing number of undocumented immigrants [12]. Needless to say, undocumented immigrants are among the most disadvantaged groups on the labour market with few options beside informal and highly precarious types of work, entirely at the mercy of the employers.

In conclusion, if we uphold the idea that the registers offer universal coverage of the population, we risk presenting a skewed picture of employment, migration and health in the Nordic countries. In a way, this development may be seen as symptomatic of a larger political transition in which the Nordic countries maintain a self-perception of a universal welfare state where every single resident is included, while, in fact, the holes in social safety nets grow larger alongside growing hidden populations. Public health researchers will need to maintain the important work of quality control and to continuously assess the validity and completeness of the Nordic registers. One crucial component of this work is to promote linkages with other types of material, including survey data [13]. Doing so will ensure that the Nordic population registers maintain their unique relevance and significance in the future.
